# White-Tailed Deer Prion Protein Gene Variability Suggests Selection Against Chronic Wasting Disease in Canada’s Prairies

**DOI:** 10.3390/v17081121

**Published:** 2025-08-15

**Authors:** William Pilot, Maria I. Arifin, Antanas Staskevicius, Nicholas J. Haley, Gordon Mitchell, Jiewen Guan

**Affiliations:** 1 National and WOAH Reference Laboratory for Scrapie and CWD, Canadian Food Inspection Agency, Ottawa, ON K2J 4S1, Canada; 2 Department of Microbiology and Immunology, College of Graduate Studies, Midwestern University, Glendale, AZ 85331, USA

**Keywords:** chronic wasting disease, prion protein gene, polymorphism, susceptibility, transmission, white-tailed deer, selection, Canada

## Abstract

Chronic wasting disease (CWD), a transmissible spongiform encephalopathy that targets cervids, has become a significant threat to both free-ranging and captive populations of Canadian white-tailed deer. In an effort to mitigate its spread, research in the past 20 years has demonstrated that the genetic background of deer may influence the pathogenesis of CWD. Specifically, variants located on the 95-, 96-, 116- and 226-codon of the prion protein gene seem to attenuate disease progression in white-tailed deer. The influence of these alleles on the likelihood of being found CWD-positive on Saskatchewan and Albertan farms was assessed using a Bayesian logistic regression model. To assess the presence of selection for favourable prion protein gene alleles, shifts in variant genotype frequencies were examined over the last seventeen years. Our results show that deer harboring the G96S allele have significantly lowered odds of infection within Canadian herds. Furthermore, the prevalence of this allele has increased significantly in farmed deer over the past seventeen years. Establishing the dynamic genetic background of Canadian deer populations will inform future disease management initiatives.

## 1. Introduction

In February of 2024, the first cases of chronic wasting disease (CWD) were detected in the province of British Columbia [1], marking the fifth Canadian province in which this fatal, cervid-specific transmissible spongiform encephalopathy (TSE) has spread [2,3]. CWD was first detected in mule deer and black-tailed deer from a Colorado research facility during the 1960s [4]. Modelling suggests that the disease was present within wildlife populations for several years despite the first wild case being confirmed in 1981 [5]. Since then, challenges with the detection and management of CWD have resulted in continued expansion into new regions. In 1996, farmed CWD cases started being detected on Saskatchewan farms, with the number of infected deer quickly increasing in the following years [6]. The detection of CWD in the Canadian population was later attributed to the import of infected cervids from the United States [6]. The spread of disease within Saskatchewan and Albertan farmed populations during the late 1990s and early 2000s coincided with the detection of CWD in free-ranging Canadian deer [5]. Recent detections in Québec, Manitoba and British Columbia, as well as widespread transmission throughout the United States of America (USA), highlight the persistent threat of CWD to cervid populations as prevalence increases.

As a prion disease, CWD is characterized by the accumulation of the infectious and pathogenic prion isoform, surnamed PrP^Sc^. The term PrP^Sc^ derives from scrapie (Sc) and can be used to denote any infectious prion formed by the misfolding of the cellular prion protein (PrP^C^), including the prions causing CWD (i.e., PrP^CWD^) [7]. These prions are typically transmitted horizontally as they are introduced either by direct interaction with CWD-infected hosts or by ingestion of infectious materials in the environment [6]. Prions excreted in feces, urine or saliva can persist in environmental soils, waters and vegetation, risking infection for years after initial deposition [8,9,10,11,12,13,14,15]. Once prions are ingested, they accumulate in the oral- and gut-associated lymphoid tissues and eventually enter the central nervous system [16,17]. CWD pathogenesis is characterized in part by the spread of prion deposits throughout lymphatic and brain tissues, particularly in the obex and retropharyngeal lymph nodes. In advanced cases of CWD, prion deposits can also be detected in other organs and peripheral tissues such as skin, muscle, heart and kidney [18,19,20]. The infectious PrP^Sc^ isoform aggregates to perturb normal central nervous system function by affecting neuronal proteostasis and causing neurotoxicity [21]. CWD infection inevitably leads to the death of the affected animal.

The conformational change from PrP^C^ to PrP^Sc^ can either occur spontaneously or be catalyzed by the seeding ability of PrP^Sc^ itself [7]. This second mechanism is what allows the amplification of PrP^Sc^ to occur within the host. Thus, as protein folding has a significant role in CWD, the protein’s primary structure is critical to pathogenesis. The prion protein gene (PRNP) has been the subject of increased research in recent years, as the gene’s polymorphisms have shown differential susceptibilities to CWD [22]. Four key polymorphisms have been identified in white-tailed deer (WTD): wild-type glutamine (Q) or histidine (H) at the 95-codon, wild-type glycine (G) or serine (S) at the 96-codon, wild-type alanine (A) or G at the 116-codon, and wild-type Q or lysine (K) at the 226-codon. None of these alleles allow for complete resistance against CWD, but they reduce the odds of infection through changes to disease onset, progression, strain selection and PrP^CWD^ kinetics [19,23,24]. Horizontal transmission to other WTD may also be reduced by the limited peripheral prion deposits and lesser fecal prion content in these variant deer [9,19]. Other PRNP polymorphisms are located at upstream promoter Affx-574071595 and the 37-, 100-, 103-, 123- and 230-codons, although these have received less attention [25,26]. Some variants found outside of the PRNP gene implicated in other TSEs and neurodegenerative diseases (e.g., Creutzfeldt–Jakob, Alzheimer’s, Parkinson’s) may also have some involvement in pathogenesis or vulnerability to CWD [26]. Our analysis focuses on the four key variants described above (p.Q95H, p.G96S, p.A116G and p.Q226K).

Wild WTD populations have shown declines associated with high CWD prevalences [27]. The important ecological and economic impacts of these losses have motivated research into genetic background as a modifying factor to CWD pathogenesis. While there is extensive research on the subject in the USA, there have been few studies focusing on Canadian populations despite the presence of the disease in the country since 1996 [3]. In their study, Wilson et al. [28] observed WTD and mule deer PRNP heterogeneity in Alberta and Saskatchewan, reporting a significant under-representation of the 96- and 185-codon variants in CWD-positive WTD populations. Later, Haley et al. [23] reported that WTD from these same provinces with certain 95-, 96-, 116- and 226-codon genotypes were less likely to be found infected. This research has been invaluable to describe PRNP variability in a Canadian context, yet it lacks the temporal scope found in some American studies [29,30]. Here, we build upon this past research by reporting on the genetic background of farmed WTD in Alberta and Saskatchewan over the last seventeen years. Included in our analysis is over 1800 individuals from twelve different herds that were sent to the World Organisation for Animal Health Reference Laboratory for CWD testing in Ottawa (Canadian Food Inspection Agency, CFIA). These deer were either genotyped at the 96-codon or sequenced along PRNP. With this genetic data, we highlight the PRNP alleles with a role in reducing the odds ratio of CWD infection in Canadian deer. In addition, we use the available information from the past seventeen years to show trends in the proportion of deer with these variants in CWD-endemic provinces.

## 2. Materials and Methods

### 2.1. Cervid Populations

The CFIA receives cervid samples for CWD initial and confirmatory testing. WTD samples have been submitted for genotyping at the 96-codon since 2007 and sequencing of the PRNP since 2019. Some samples received after 2019 were both genotyped and sequenced, and alignment between the two results was assured. A subset of 1834 WTD samples received by the CFIA between the years 2007 and 2024 were retrospectively used for analysis in this study (Table 1). This subset contains samples sourced from herd depopulations in Alberta (*n* = 1342) and Saskatchewan (*n* = 492). All of the animals included in this study were sourced from herds exposed to CWD, although the level of exposure could not be controlled, which is a limitation of the study design. A variety of tissues such as retropharyngeal lymph node (RPLN), brain, blood and skin were used for DNA extraction. Both RPLN and brainstem homogenates were subjected to diagnostic testing by enzyme-linked immunosorbent assay (ELISA) using the TeSeE SAP COMBI Kit (Bio-Rad, cat #3551191, Marnes-la-Coquette, France), as per the manufacturer’s protocol. Positive ELISA results were confirmed by immunohistochemistry (IHC). Out of the 1834 animals tested using ELISA, 1776 had available 96-codon genotype and 800 had available PRNP sequences. These 1834 animals were used to calculate the odds ratios. A subsequent temporal study of 96-codon variation included additional CFIA-collected deer previously reported by Haley et al. [23] (Table 1). These were extra deer for herd E and herds M through U, for a total of 1164 additional genotyped individuals included only in the temporal analysis. While these deer were not included in the calculation of the odds ratio, they were used to ensure farm-wide CWD prevalence was accurate.

### 2.2. DNA Extraction, Real-Time PCR Genotyping and Sanger Sequencing

Tissue or blood samples were extracted using the automated QIAcube HT instrument (Qiagen) and QIAamp 96 DNA QIAcube HT Kit (Qiagen, cat #51331, Hilden, Germany) or manually extracted using the DNeasy Blood and Tissue Kit (Qiagen, cat #69504, Germantown, MD, USA). Extracted DNA was stored at −20 °C or immediately used for downstream analyses.

For real-time TaqMan-based genotyping, cervid PRNP was first amplified by conventional PCR from a minimum of 10 ng DNA using the indicated primer set and a modified protocol from O’Rourke et al. [31]. Briefly, primary amplification of the entire open reading frame of the cervid PRNP on exon 3 was performed using the 223-forward (5′-ACACCCTCTTTATTTTGCAG-3′) and 224-reverse (5′-AGAAGATAATGAAAACAGGAAG-3′) primers. The protocol is as follows: initial denaturation at 94 °C for 3 min, 15 cycles of denaturation at 94 °C for 30 s, annealing at 54 °C for 30 s and elongation at 68 °C for 90 s, with a final extension at 68 °C for 5 min. The detection of alleles at the 96-codon was performed using a real-time TaqMan-based assay, in which the primary amplicons were diluted 1:100 then subjected to amplification using forward primer 5′-AGCCACATGGTGGTGGAG-3′ and reverse primer 5′-TGGTTTACTGGGCTTGTTCC-3′. Probes 5′-FAM-TCAAAGTGGTACCCACAG-MGB-3′ and 5′-HEX-TCAAGGTGGTACCCAC-MGB-3′ were used to detect alleles G and S, respectively. The real-time PCR protocol was as follows: initial denaturation at 95 °C for 3 min, followed by 40 cycles of denaturation at 95 °C for 20 s and annealing–elongation at 60 °C for 40 s. Data analysis and allelic determination was completed using the CFX Maestro version 2.3 software (Bio-Rad).

For Sanger sequencing, primary amplification was performed as mentioned above [31] from a minimum of 10 ng DNA. This was followed by a dye terminator reaction using the BigDye^®^ Terminator v3.1 Cycle Sequencing Kit (Life Technologies, cat# 4337457, Warrington, UK), with slight modifications to the manufacturer’s protocol. Briefly, forward (5′-GGGCTCGAGGTCATCATGGTGAAAAGCCACATAGG-3′) and reverse (5′-CCCACGCGTCTATCCTACTATGAGAAAAATGAGG-3′) gene-specific primers were used to sequence each sample, and the 2.5X BigDye^®^ Terminator reagent (Life Technologies, cat# 4337457, Warrington, UK) was diluted 1:17 in approximately 6% trehalose, 1X Sequencing Buffer and molecular-grade water. The cycling conditions were as follows: initial denaturation at 96 °C for 1 min, followed by 25 cycles of denaturation at 96 °C for 1 min, annealing at 52 °C for 5 s and elongation at 60 °C for 4 min. Excess labelled nucleotides were removed using the BigDye^®^ XTerminator Kit (Life Technologies, cat #4376487, Bedford, MA, USA). Samples were submitted for Sanger sequencing to our in-house Central Sequencing Unit, Ottawa Laboratory Fallowfield. Sequences were analyzed using MEGA version 11 or Geneious Prime version 2023.2.1.

### 2.3. Data Analysis

Data analysis was completed on RStudio version 4.3.1. A Bayesian mixed-effects logistic regression modelling the risk of a CWD-positive test with 95-, 96- and 116-codons as predictor variables was performed using the brms package [32,33]. Only common alleles (over 1%) were included in the model, resulting in the removal of the 226-codon. The 95- and 116-codon combined variant homozygotes with heterozygous WTD due to very low homozygous counts (95*H and 116*G genotypes). For all three codons, the wild-type genotype was used as reference. To account for prevalence and genetic variation within herds, herd was included as random effect. Model coefficients were attributed as being weakly informative prior to the Student T distribution, with degree of freedom 5, center 0 and a scale parameter of 2.5 [34]. We performed Markov chain Monte Carlo sampling at 100,000 iterations after an initial warmup of 10,000. This was run four separate times (four chains), resulting in potential scale reduction factors of 1 and effective sample sizes ranging from 80,000 to just over 340,000. The potential scale reduction factor is a measure of chain convergence, with values surrounding 1 indicating good chain mixing [35]. The effective sample sizes are measures of information content, with higher counts indicating higher reliability of the estimates [35,36]. Model coefficients (*β* values) represent the natural logarithm of the odds ratio, and so odds ratios were calculated by exponentiating the coefficients.

We also assessed the likelihood of infection by comparing both genotype and allele distributions to CWD status with chi-square test and Fisher’s exact test for count data. To specify significant differences between cells in contingency tables bigger than 2 × 2, standardized residuals from chi-square tests were used [37]. A difference from the expected value was considered significant when the absolute value of the standardized residuals exceeded 2.6. This value is derived from Bonferroni’s adjustment to the significance level according to the number of cells in the 3 × 2 contingency tables [37]. For example, the CWD-positive 96GS cell has a standardized residual of −3.57, thus there is significantly less CWD-positive 96GS deer than expected.

Linear models were also created to assess temporal changes in genotype frequency. Data included both current and previously reported Canadian data to allow for a wider temporal analysis [23]. The frequency of 96-codon genotype was modeled against two interacting variables as follows:

*y* = *β*_0_ + *β*_1_*X*_1_ + *β*_2_*X*_2_ + *β*_3_*X*_1_*X*_2_, (1)

where *X*_1_ is the amount of time CWD had been present in the province and *X*_2_ is the rate of CWD in the herd. This would account for the difference in length and strength of potential selective pressure. Frequencies were weighted according to the total number of WTD in the herd. Model coefficients (*β* values) represent the change in genotype frequency with either time or herd prevalence (when the other is held constant). The number of years since the first farmed CWD case was calculated by subtracting 1996 or 2002 from the submission year, depending on if the animal was from Saskatchewan or Alberta, respectively. Herd prevalence was determined from farm-wide test results.

## 3. Results

We determined the 96-codon genotype of 1776 WTD using real-time PCR or sequencing (Table 2). The 96-codon genotype differed from the wild-type in 41.22% of individuals. Additionally, we reviewed the sequences of the PRNP from 800 WTD. The most common PRNP was expectedly the wild-type PRNP consisting of 95QQ, 96GG, 116AA and 226QQ. Polymorphism at all four observed regions was detected, with the most common polymorphism being at the 96-codon, with the variant G96S allele. Among only the animals with a full PRNP sequence, variation at the 95-codon was found in 3.88% of WTD, in 34.64% of WTD at the 96-codon and in 3.01% of WTD at the 116-codon. Polymorphism of the 226-codon was not detected. Its percentage may be slightly underestimated due to the presence of 20 incomplete sequences missing the 226-codon genotype. Table 3 shows the remaining samples with complete 95-, 96- and 116-codons. Some of the animals harbored two variants, although phase information could not be determined. No animals harboring the Q95H allele and only one animal with an A116G allele were found in Saskatchewan.

Logistic regression using these farmed animals revealed reduced odds of infection for CWD in WTD with the G96S allele. It was found that 96GS deer have 45.4% lower odds (*β* = −0.61, 95% CI: −1.30–0.07) and 96SS deer have 96.6% lower odds (*β* = −3.38, 95% CI: −8.82–−0.44) of being found CWD-positive, when compared with wild-type WTD. However, the Bayesian credible interval of the 96GS genotype overlaps with 0. The reduced odds from the 116*G genotype (*β* = −0.48, 95% CI: −1.66–0.64) also overlap 0, although to a greater extent. These overlaps are important as the odds ratio would then overlap 1, meaning the odds ratio of infection may not be significantly different to the reference wild-type deer. Deer with the Q95H allele had increased odds of infection (*β* = 0.76, 95% CI: −1.15–2.63), but once again at a non-significant level. Higher animal counts could feasibly reduce error and supply more precise results for all three codons.

Comparisons between genotypes and alleles using chi-square and Fisher’s exact tests support the above findings. The 96-codon had significant differences with both tests in the allele distribution between positive and negative WTD (Table S1). Animals harboring the G96S variant were under-represented in positive animals, suggesting some form of fitness advantage against CWD. Much like the regression results, there were no significant shifts in allele distribution for any of the other codons. Looking more closely at the 96-codon, there are also differences with both tests in the genotype distribution of these animals across CWD status (Table S2). In the CWD-positive group, there was a significant over-representation of 96GG and a significant under-representation of both 96GS and 96SS animals (Table S3). As such, despite the above model’s non-significant result for 96GS deer, these two tests suggest that the G96S allele is pertinent to the odds of infection. Both genotypic and allelic frequencies of the 95- or 116-codon do not show significant differences between CWD-positive and -negative animals.

**Figure 1:**
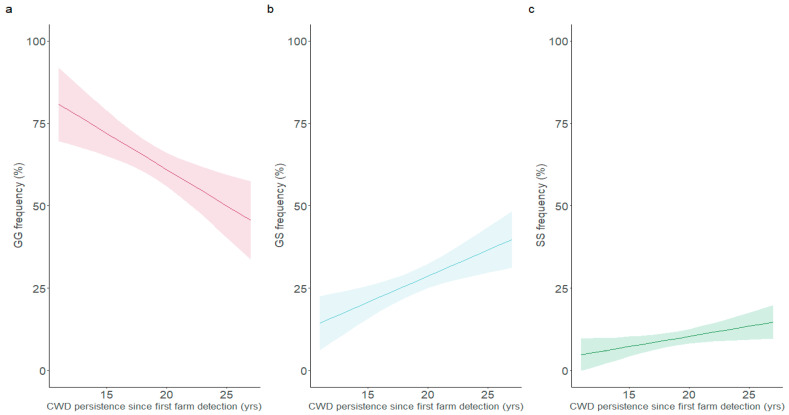
Linear regression modelling changes in the frequency of 96GG (**a**), 96GS (**b**) and 96SS (**c**) animals according to the number of years CWD has been detected in the province’s farmed population. The model inputs were 46 herd year points from 21 different herds between 2007 and 2024, including herd data pulled from Haley et al. [23]. The lighter area around the fitted line represents the 95% confidence intervals.

**Figure 2:**
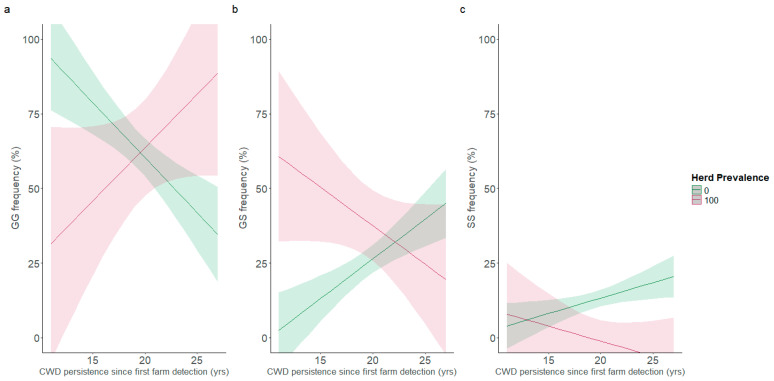
Interaction plots of 96GG (**a**), 96GS (**b**) and 96SS (**c**) genotype frequency according to the number of years CWD has been detected in the province’s farmed population at predicted herd prevalence extremes. The model inputs were 46 herd year points from 21 different herds between 2007 and 2024, including herd data pulled from Haley et al. [23]. The lighter area around the fitted line represents the 95% confidence intervals.

The rest of the analysis was focused on the 96-codon, as it was the only region of interest highlighted significantly in the models above. It also had the most available data, with analysis starting in 2007, as opposed to the other three key polymorphisms’ analysis starting in 2019. Linear regressions revealed significant temporal shifts in the distribution of 96-codon genotype, in favour of the more protective variants (Figure 1a–c). The decrease in susceptible genotype frequency attributed only to time since the detection of CWD (pressure length) was approximately 3.69% per year (*β*_1_ = −3.69, *p* < 0.001). Conversely, the regression showed a decreasing 96GG population (*β*_2_ = −1.42, *p* = 0.01) as herd prevalence (pressure strength) increased. While this could suggest that 96GG deer are uncommon in high prevalence herds, the model also contains a positive interaction effect incorporating both the pressure length and strength variables (*β*_3_ = 0.07, *p* = 0.01). In 2025, 29 years since the first CWD case in Saskatchewan and 23 years since the first CWD case in Alberta, this interaction effect would indicate a 2.03% or 1.61% increase in 96GG deer per 1% increase in herd prevalence, respectively. These outweigh the pressure strength effect, but not the pressure length effect in cases where herd prevalence is low, signaling an increase of 96GG frequencies exclusively in high CWD prevalence herds. Thus, while 96GG deer were common in the past, the predicted frequency of the 96GG genotype is diminishing with time in low prevalence herds (Figure 2a). The opposite trends are being found in 96GS and 96SS deer. A corresponding increase of 2.65% (*β*_1_ = 2.65, *p* < 0.001) and 1.04% per year (*β*_1_ = 1.04, *p* = 0.02) attributed only to time in 96GS and 96SS deer, respectively, is detected. The pressure length coefficient is the only significant coefficient in the 96SS genotype frequency model. However, the 96GS model contains a significant negative interaction effect (*β*_3_ = −0.05, *p* = 0.01) and a significant positive pressure strength effect (*β*_2_ = 1.16, *p* = 0.005). In 2025, this interaction effect (−1.52% per 1% herd prevalence in Saskatchewan and −1.20% per 1% herd prevalence in Alberta) would also outweigh the pressure strength effect but not the pressure length effect when prevalence is low. Thus, both 96GS and 96SS are increasingly being detected, and 96GS are being over-represented in low prevalence herds (Figure 2b).

## 4. Discussion

In the present study, samples from 1834 WTD were retrospectively analyzed for odds ratio analysis. The 96-codon genotype of 1776 deer and the sequencing of a subset of 800 deer have shown the presence of Q95H, G96S and A116G variants on Albertan and Saskatchewan farms. Although the G96S allele was found at a decidedly higher frequency of 41.22%, we report Q95H and A116G alleles in 3.88% and 3.01% of WTD, respectively. Studies reporting on Canadian farmed populations show similar levels of 96-codon variants, although earlier studies using free-ranging deer suggest these high levels may have been present in wildlife as early as 2009 [23,28]. The very low counts of other variant deer on Canadian soil have unfortunately made it difficult to assess their influence on the likelihood of being found CWD-positive. The frequency of WTD carrying the Q95H allele has seemed to consistently remain around 2–4% for the past 15 years, while WTD with the A116G allele are found at a slightly higher level of approximately 6–8% [23,28]. Conversely, the levels of 96GS or 96SS WTD in the USA are generally much lower at around 15–20%, although one study has shown it has the potential to reach levels similar to the Canadian population [23,38,39,40,41,42]. However, reports from the USA show that there are comparable levels of 95*H WTD to Canadian populations, well below 10% [23,38,39,40,41,42]. The A116G allele is rarely, if ever, reported in American studies despite its presence in Canada for at least fifteen years. The opposite is true for the Q226K allele, whose presence has yet to be reported in Canadian studies [23,28]. However, unpublished data from the CFIA confirm their emergence in Albertan and Manitoban wildlife between 2020 and 2024, albeit at a very low frequency. As the most common variant in the population, the G96S allele is known to reduce the risk of being found CWD-positive. However, there are substantiated concerns about the consequences of delaying visible disease onset and CWD endpoint if such deer were to become infected. Thus, disease management may become a balancing act between the potential to reduce the unparalleled spread of CWD, and the risks of unpredictable strain emergence and PrP^CWD^ deposition across North American ecosystems.

In this study, logistic regressions show reduced odds of infection in 96SS WTD and to a lesser extent in 96GS WTD. Accordingly, chi-squared and Fisher’s exact comparison tests showed that both animal genotypes were significantly under-represented among CWD-positive deer. As such, both 96GS and 96SS WTD are less likely to be found infected in Canadian herds. This finding is echoed across many different studies associating the lower rates of infection with these genotypes in both Canadian and American populations [23,38,39,40,41,42]. A 2022 genome-wide association study on the genome of WTD has highlighted polymorphism of the 96-codon as having the largest effect on the susceptibility to CWD [26]. Importantly, the G96S allele increases survival times for deer, potentially through the extending the incubation period of CWD [22]. It is therefore possible that the 96GS and 96SS populations analyzed in this study were simply at an early stage of CWD before being depopulated, and that these deer would have gone on to become CWD-positive. The other three rarer variants were not found to be significantly under-represented in infected populations, despite previous studies showing they lower the odds of infection in North American deer [23,38,39,40]. While polymorphism at the 116-codon did in fact lead to an odds ratio below 1, the 95% Bayesian credible interval did not allow us to conclude that this reduction is significant. Additionally, the chi-squared and Fisher’s exact comparison tests do not show significant under-representation in CWD-positive animals of any genotype other than 96GS and 96SS. This is likely due to the persistent rarity of certain alleles, a problem that has plagued other similar studies. The lowered odds ratio of infection in G96S variant deer may be explained by the structural and functional differences during prion misfolding. Biomolecular analyses revealed that 96S prions may be less structurally stable and compact than wild-type prions [43]. It also possesses significantly weaker seeding activity during the cellular prion misfolding reaction [24]. This reduced efficiency of prion amplification in the deer would lead to slower disease progression, with lesser IHC staining in the obex and increased incubation times [23,44,45]. So, WTD with modifications to the prion protein are not only less likely to become infected with CWD, but they will also succumb to the disease later than susceptible deer. This may increase opportunities to pass on the protective alleles to future generations, potentially increasing the frequency of 96GS and 96SS deer in Canadian populations. It is important to note that these changes to typical CWD pathogenesis do not translate into complete immunity to the disease. Multiple 96GS and 96SS animals were found to be infected. CWD was also detected in 95QH and 116AG animals, even when these animals also harbored a G96S allele.

There are also important considerations against artificially selecting for these variant deer. CWD infection in these animals sometimes leads to PrP^CWD^ conformations with unique properties. For example, deer with the Q95H allele shed the H95^+^ strain of infectious prions, capable of infecting tg60 mice (G96S allele) more efficiently than the wild-type Wisc-1 CWD prion strain [46]. More research into strains is warranted to avoid the emergence of prion strains with aggravated pathogenesis, increased infectivity or a weaker species barrier. Next, environmental prion deposition as a direct consequence of host–host and host–environment interactions during disease courses would be extended when animals do not succumb to CWD in a timely manner. In fact, despite their partially protective alleles, variant deer still accumulate PrP^CWD^ activity in the intestine, bladder and salivary glands, resulting in shed prion content through excreta [19,47,48,49]. Critically, however, the reduction in peripheral accumulation of PrP^CWD^ in variant deer may partly attenuate this effect in white-tailed deer with two variant alleles [19,47,48,49]. As these pre-clinical deer age and accumulate prion content, the older deer would become more vulnerable to predation and parasitism [50,51]. Coyotes fed on infected cervid brain meal and parasites (nasal botflies and black-legged ticks) fed on infected blood meal were found to have detectable PrP^CWD^ content in their excreta, exposing potentially important routes of natural infection [52,53,54]. More research would be necessary to effectively characterize the differences in prion shedding between WTD of different genotypes. Additionally, intensive breeding as a disease control method has been shown to lead to considerable genetic variability loss [55]. This may overall reduce the general population’s fitness in and of itself.

To evaluate the presence of an overall shift in genotypic proportions, the frequency of 96GS and 96SS deer was calculated at multiple herd year points. It was found that not only was the G96S allele the most common variant in the Canadian farmed WTD population, but there was a clear increase in its proportion from 2007 to 2024. Additionally, these deer are being increasingly found in Canadian farmed populations with a lower CWD prevalence. These findings echo trends from previous experiments applying selective breeding programs to farmed WTD populations [56]. Evidence of this selective pressure is reported across cervid species in both farmed and wildlife populations [29,30,57]. As both statistical and biomolecular experiments have shown the advantage variant deer hold against wild-type deer, it is reasonable to believe that this increased fitness may lead to some form of pathogen-driven selection. However, the genetics of farmed deer populations are often heavily influenced by artificial selection for a range of production-related traits. Shortly after studies began identifying a role for the G96S variant in CWD, some producers began incorporating this as a factor in their breeding programs. Controlled selection for these more resistant genotypes has been shown to abruptly decrease the frequency of wild-type WTD [56]. Unfortunately, the timing and extent to which this occurred in Canadian deer herds is difficult to pinpoint, although it is highly likely that artificial selection played a major role in any trends in genotypic frequency. These results demonstrate some form of selection in effect for the G96S allele in Canadian endemic regions, likely to be strongly influenced by producer activities. Importantly, while the model accounts for herd-level differences in CWD exposure, practices such as separating the variant deer or differences in care could lead to unaccounted within-herd variation. As a result, producer activities may act as a confounding factor and limit the accuracy of our analysis.

The implications of these trends are not only important for disease management in captive herds, but also for wildlife disease control. Farmed populations can be used to study infectious disease dynamics in wildlife when population dynamics and spatial elements are similar [58]. Chronic wasting disease and intense hunter harvest are the leading causes of mortality in both ranches tailored to big game hunting and endemic regions, suggesting that the selective pressures on WTD may not be too dissimilar [27,59]. While it may not be as intense, it is still reasonable to expect shifts in 96-codon genotype frequency in Saskatchewan and Albertan wildlife similar to what is seen in the above farms. American mule deer, white-tailed deer and elk free-ranging population dynamics suggest that the variant alleles (S225F, G96S and M132L, respectively) are already being favoured in response to CWD spread [29,30,57]. These variants are associated with delays in CWD pathogenesis, leading to more opportunities to pass protective alleles to offspring [29,30,60]. While there are barriers to deer movement across farms, no such obstacles exist in the wild. The migration of variant deer across state, province and national borders is less controlled, and so natural selection observed in the USA could be reflected in Canadian wildlife populations as well. Furthermore, the environmental deposition of prions and rare direct contacts at farm fence lines may contribute to disease transmission both to and from wildlife [61]. Many species of deer, including WTD, have been shown to frequent regions directly besides fence delimitations often when in captivity [61,62]. This increases the potential for the direct and indirect horizontal transmission of CWD between captive and free-ranging animals. In these cases, the relative susceptibilities to CWD attributed to PRNP genotypes could have a significant role in reducing spread both in captivity and in the wild. Thus, regions with high frequencies of variant deer may appear more resistant to the spread of CWD. With continuous spread to new provinces and states, such areas may act as barriers to slow transmission across the continent. This is especially important as currently no such barriers exist to reduce the Canadian spread of CWD [3]. Regions that have been shown to carry low counts of G96S allele (or other) deer may be labelled as especially susceptible to CWD. This would allow authorities to specifically align disease management initiative severity with risk of spread. Further sequencing of pre-2019 Canadian samples for information on the 95-,116- and 226-codons would also allow more detailed temporal analyses of shifts across the Canadian landscape. Continuous monitoring and reporting of WTD PRNP polymorphism might prove to be an important tool to wildlife and regulatory bodies in handling the increasing threat of CWD, as well as deepening the understanding of their role in infectious disease dynamics.

## Figures and Tables

**Table 1:** Overview of the 21 herds included in the study. Herds E and M-U are italicized as they include previously reported animals by Haley et al. [23]. For herd E, the previously reported animal count is placed in brackets. The herds were depopulated from Saskatchewan or Albertan farms between 2007 and 2024. Herd prevalences were calculated from official CWD testing results of the depopulated herds. The number of animals per herd is shown alongside the number of WTD and the proportion of WTD samples having been genotyped at the 96-codon (2007–2024) or PRNP sequenced (2019–2024). Herds were anonymized for privacy. “AB” = Alberta, “SK” = Saskatchewan. “ND” = not done.

Herd	Province	% CWD (Farm-Wide)	n	n_WTD_	n_Genotyped_	n_Sequenced_
A	AB	3.70	378	378	377	313
B	AB	0.00	306	306	ND	296
C	AB	11.89	143	136	136	ND
D	AB	4.66	686	402	388	29
*E*	SK	64.90	126 (+312)	438	71 (+312)	108
F	SK	1.09	274	274	271	ND
G	SK	11.77	20	12	12	ND
H	SK	90.00	20	20	18	9
I	SK	67.74	31	34	34	ND
J	SK	11.36	44	86	24	ND
K	SK	8.33	96	96	96	52
L	SK	85.71	21	31	31	ND
*M*	SK	31.40	121	24	24	ND
*N*	SK	6.7	179	157	157	ND
*O*	SK	47.62	23	21	21	ND
*P*	SK	21.26	414	395	381	ND
*Q*	SK	7.58	66	36	36	ND
*R*	SK	82.76	29	29	29	ND
*S*	SK	23.21	56	56	56	ND
*T*	SK	69.74	76	69	69	ND
*U*	SK	18.06	72	58	55	ND

**Table 2:** Animal count and CWD status of WTD according to 96-codon genotype. All twelve herds had available 96-codon genotype data. Wild-type deer express the 96GG prion protein. Herds range from 2007 to 2024. Total counts and frequency are also shown. * Animals were not found in this herd.

Herd	Wild-Type		96GS		96SS	
	CWD+	CWD−	CWD+	CWD−	CWD+	CWD−
A	13	270	1	65	*	29
B	*	164	*	67	*	65
C	16	64	1	41	*	14
D	10	179	2	136	*	75
E	59	27	16	9	*	1
F	2	154	*	86	1	28
G	3	6	*	3	*	*
H	16	1	1	2	*	*
I	8	2	6	3	*	*
J	4	5	1	3	*	*
K	4	18	3	53	1	17
L	18	1	*	2	*	*
Total	153	891	31	470	2	229
*f* (%)	58.78		28.21		13.01	

**Table 3:** Animal count and CWD status of WTD according to 95-, 96- and 116-codon genotypes. Six of the twelve herds had available sequencing data. Herds range from 2019 to 2024. Wild-type deer express the 95QQ, 96GG and 116AA prion protein. When an animal is found to have more than one variant, they are both included and separated by a “/”. Total counts and frequency are also shown. * Animals were not found in this herd.

Herd	Wild-Type		96GS		96SS		116AG		116GG		95QH		95HH		96GS/ 116AG		95QH/ 96GS	
	CWD +	CWD −	CWD +	CWD −	CWD +	CWD −	CWD +	CWD −	CWD +	CWD −	CWD +	CWD −	CWD +	CWD −	CWD +	CWD −	CWD +	CWD −
A	13	211	1	49	*	22	*	7	*	4	*	1	*	*	*	*	*	4
B	*	145	*	63	*	65	*	*	*	*	*	9	*	10	*	*	*	4
D	8	8	2	7	*	*	1	1	*	*	*	*	*	*	*	2	*	*
E	47	22	13	9	*	1	3	4	*	*	2	*	*	*	1	*	1	*
H	6	1	2	*	*	*	*	*	*	*	*	*	*	*	*	*	*	*
K	1	18	1	20	*	10	1	*	*	*	*	*	*	*	*	*	*	*
Total	75	405	19	148	0	98	5	12	0	4	2	10	0	10	1	2	1	8
*f* (%)	60.00		20.88		12.25		2.13		0.50		1.50		1.25		0.38		1.13	

## Data Availability

The data presented in this study are available on request from the corresponding author due to privacy considerations. The RStudio scripts used to perform this study’s data analysis are available online at https://github.com/wilpilot/WTD-PRNP-Variability (accessed on 3 June 2025).

## Supplementary Materials

The following supporting information can be downloaded at: https://www.mdpi.com/article/10.3390/v17081121/s1, Table S1: Allele counts (*n*) and frequencies (*f*) of the 1776 WTD samples submitted to genotyping and/or sequencing; Table S2: Genotype counts (*n*) and frequencies (*f*) of the 1776 WTD samples submitted to genotyping and/or sequencing; Table S3: Standardized residuals from the genotype 2 × 3 Chi-square tests.

## Abbreviations

The following abbreviations are used in this manuscript:

CFIA	Canadian Food Inspection Agency
CWD	Chronic wasting disease
ELISA	Enzyme-linked immunosorbent assay
IHC	Immunohistochemistry
PRNP	Prion protein gene
PrPC	Prion protein (cellular)
PrPCWD	Prion protein (infectious, chronic wasting disease)
PrPSc	Prion protein (infectious, general or scrapie)
RPLN(s)	Retropharyngeal lymph node(s)
TSE	Transmissible spongiform encephalopathy
USA	United States of America
WTD	White-tailed deer
