# Supplementary material for: White-Tailed Deer Prion Protein Gene Variability Suggests Selection Against Chronic Wasting Disease in Canada’s Prairies
Source: Viruses. 2025 Aug 15;17(8):1121. doi: 10.3390/v17081121 (PMC12390674; doi:10.3390/v17081121)
Supplement: Supplementary file 1 [file viruses-17-01121-s001.zip › viruses-3737099-supplementary.pdf]

Allele	n	f	CWD+	CWD-	P-values
		**Codon 95**			
**Q**	1563	97.4	203	1360	0.28 (0.35)
**H**	41	2.6	3	41	
		**Codon 96**			
**G**	2589	72.9	338	2251	<0.001 (<0.001)
**S**	963	27.1	36	927	
		**Codon 116**			
**A**	1574	98.3	200	1374	0.17 (0.16)
**G**	28	1.7	6	22	
		**Codon 226**			
**Q**	1560	100.0	202	1358	NA
**K**	0	0.0	0	0	

Table S1: Allele counts (*n*) and frequencies (*f*) of the 1776 WTD samples submitted to genotyping and/or sequencing. The amount of allele copies is associated to CWD status and evaluated by Chi-square and Fisher's exact tests. Chi-square test results are shown, and Fisher's exact test results are inserted in brackets. "NA" = not applicable

Genotype	n	*f*	CWD+	CWD-	P-values
		**Codon 95**			
**QQ**	770	96.1	100	671	
**QH**	21	2.6	3	18	0.53 (0.62)
**HH**	10	1.2	0	10	
		**Codon 96**			
**GG**	1044	58.8	153	891	
**GS**	501	28.2	32	469	<0.001 (<0.001)
**SS**	231	13.0	2	229	
		**Codon 116**			
**AA**	777	97.0	97	680	
**AG**	20	2.5	6	14	0.08 (0.08)
**GG**	4	0.5	0	4	
		**Codon 226**			
**QQ**	780	100.0	101	679	
**QK**	0	0.0	0	0	NA
**KK**	0	0.0	0	0	

Table S2: Genotype counts (*n*) and frequencies (*f*) of the 1776 WTD samples submitted to genotyping and/or sequencing. The amount of genotype copies is associated to CWD status and evaluated by Chi-square and Fisher's exact tests. Chi-square test p-values are shown, and Fisher's exact test p-values are inserted in brackets. "NA" = not applicable

Genotype	CWD+	CWD-	P-values
	**Codon 95**		
**QQ**	0.5	-0.5	
**QH**	0.2	-0.2	0.53 (0.62)
**HH**	-1.2	1.2	
	**Codon 96**		
**GG**	6.8	-6.8	
**GS**	-3.6	3.6	<0.001 (<0.001)
**SS**	-5.1	5.1	
	**Codon 116**		
**AA**	-1.8	1.8	
**AG**	2.3	-2.3	0.08 (0.08)
**GG**	-0.8	0.8	

Table S3: Standardized residuals from the genotype 2x3 Chi-square tests. Absolute values over 2.6 represent a significant shift from the expected values (either an over- or under-representation). Chi-square test and Fisher's exact test p-values are shown again to highlight codons with a significant overall deviation from expected values.
